# Mid-term assessment of subtalar arthroereisis with Talar-Fit implant in pediatric patients with flexible flatfoot and comparing the difference between different sizes and exploring the position of the inserted implant

**DOI:** 10.3389/fped.2023.1258835

**Published:** 2023-10-02

**Authors:** Huan-guang Xie, Li Chen, Xiang Geng, Chen Wang, Chao Zhang, Xu Wang, JiaZhang Huang, Xin Ma

**Affiliations:** Department of Orthopedic Surgery, Huashan Hospital, Fudan University, Shanghai, China

**Keywords:** flexible flatfoot, outcomes, position, size, subtalar arthroereisis

## Abstract

**Background:**

Subtalar arthroereisis (STA) has gained growing acceptance as a viable approach solution for the management of pediatric flexible flatfoot. However, STA still remains controversial. The purpose of this study is to assess the effect of STA using the Talar-Fit implant for treating pediatric flexible flatfoot. Specifically, the aims of the study are as follows: first, to present the mid-term outcomes of STA using the Talar-Fit implant; second, to compare the radiographic and clinical outcomes associated with varying sizes of Talar-Fit implant; and third, to analyze the optimal position of the inserted implants.

**Methods:**

A retrospective analysis was conducted on a cohort of 57 pediatric patients diagnosed with flexible flatfoot (77 feet) who underwent STA utilizing Talar-Fit between January 2014 and December 2021. The participants were categorized into five groups according to the size of the implant: Group 8, Group 9, Group 10, Group 11, and Group 12. The evaluation included the assessment of clinical function using the American Orthopaedic Foot and Ankle Society (AOFAS) ankle–hind foot score, as well as the assessment of radiographic data such as the calcaneal pitch angle (CPA), lateral Meary angle (LMA), talar declination angle (TDA), and medial longitudinal arch angle (MLAA) were evaluated. Furthermore, the position of the inserted implants was also recorded, including angle, depth, and distance. The comparison of pre- and postoperation was conducted using the paired Student's *t*-test, whereas the analysis of differences among subgroups was performed using the Wilcoxon rank-sum test. A *P*-value < 0.05 is considered statistically significant.

**Results:**

In total, 57 pediatric patients (77 feet) were successfully followed-up for an average period of 26.8 months. The overall AOFAS score significantly improved from 58.6 ± 10.9 to 85.2 ± 8.6 (*P* < 0.001). Furthermore, the LMA decreased from 20.3° ± 3.6° to 4.5° ± 1.3°, the CPA increased from 14.8° ± 1.6° to 23.6° ± 2.7°(*P* < 0.05), the TDA decreased from 40.2° ± 2.3° to 25.5° ± 3.2°(*P* < 0.05), and the MLAA decreased from 140.1° ± 2.8° to 121.4° ± 3.9°(*P* < 0.05). No statistically significant differences were observed among subgroups regarding the final outcomes. The improvements of CPA, TDA, and MLAA among different groups were significantly different; however, the adjusted *P*-values were all greater than 0.05. The implant were inserted at a mean angle of 89.5° ± 2.4°, a mean depth of 0.9 mm ± 2.1 mm, and a mean distance of 9.9 mm ± 0.9 mm. Eight patients experienced complications, including six cases of pain occurrence and two cases of implant dislocation.

**Conclusion:**

STA with Talar-Fit has demonstrated satisfactory mid-term outcomes. A Talar-Fit with a larger size may demonstrate a superior effect when compared with that of a smaller size. The implants were inserted in a similar position, indicating that the medial edge of the implant may be possible to transcend the midline of the talus neck.

## 1. Introduction

Flexible flatfoot (FFF) is a prevalent orthopedic condition in clinical settings, particularly among the pediatric population, with a reported incidence rate of 5% (1). However, the etiology and pathological process of flexible flatfoot remain unclear (2). Flexible flatfoot is often characterized by the depression of the medial longitudinal arch, abduction of the forefoot, and eversion of the hindfoot, which may be caused by the excessive eversion of the subtalar joint (3). Other features, such as spasm of the peroneus and contracture of the gastrocnemius aponeurosis or Achilles tendon, are usually observed either individually or in conjunction with all of the aforementioned factors. Moreover, the majority of patients are often asymptomatic with a normal medial longitudinal arch. However, the main characteristics become more evident in weight-bearing, and some patients also suffer from subjective symptoms such as foot or ankle pain, gait changes, clumsiness, and easy fatigue after standing for long periods of time or walking long distances.

Treating flexible flatfoot still remains controversial (4). If not treated appropriately, some sequelae may occur, such as developing rigid flat feet and osteoarthritis, which can potentially destroy the mechanical axis of the lower limb (5). Most researchers recommend that patients exhibiting mild symptoms generally should be treated with conservative measures, such as utilizing orthopedic shoes or arch pads, while patients with persistent symptoms (such as persistent pain, gait changes, and easy fatigue) (6) should consider surgical treatment. Surgical procedures include subtalar arthroereisis (STA), osteotomies, arthrodesis, and soft tissue surgeries including tendon lengthening and transfer (7, 8). Currently, subtalar arthroereisis is gaining popularity in clinical settings due to its notable advantages of simple operation, less trauma, and better prognosis (9). STA refers to the insertion of implants into the sinus tarsi to correct excessive pronation of the subtalar joint and sustain its neutral position, consequently correcting the deformity of the flatfoot. Chambers first introduced the procedure in 1946, which gained popularity in the 1970s (10, 11). Through clinical observation, Fernández de Retana pointed out that the STA is indicated four or more of the following criteria are met: (1) no radiographic or clinical improvement after 2 years of conservative treatment; (2) valgus angle of hindfoot >10°; (3) FFF combined with Achilles tendon contracture; (4) Viladot footprint type II, III, or IV; (5) Meary angle <10°; (6) Moreau–Costa–Bartani angle >130° or Kite angle >25° (12). A wide variety of implants are now available on the market as a result of technological improvements. Vogler classified them into three types in 1987 based on their biomechanical characteristics: axis-altering devices, impact-blocking devices, and self-locking wedges. Among these types, self-locking wedges are the most commonly used in clinical practice (8). In 2012 (13), Graham and Jawrani further divided the self-locking wedge type into three types: type IA, type IB, and type II. The difference among these three types lies in the geometric design and the specific location of implantation for the implants. Type IA is designed in a cylindrical geometry, while type IB is characterized by a conical shape. Notably, both of these structures are inserted into the tarsal sinus in a lateral to medial orientation. Type II is featured with a medial-cylindrical and lateral-conical geometry, and inserted in an orientation from anterior-lateral-distal to posterior-medial-proximal. The representative implants include MBA, Talar-Fit, and HyproCure, which are widely recognized and routinely employed in clinical practice at present.

However, STA still remains controversial (14). Using the Talar-Fit as an example, the provided guidelines recommend inserting the screws into the tarsal sinus; however, many researchers prefer to insert implants into the sinus tarsal canal along the longitudinal axis of the sinus tarsal canal, anchoring them in the sinus tarsal canal (15). As a consequence, there is no uniform standard regarding the position and orientation of implants following modification. In a study conducted by Wang et al. (16), a comparison was made between two different types of devices, with two different insert positions, in order to assess the ability to correct the flatfoot deformity. The study utilized a cadaveric flatfoot model and found that HyproCure exhibited greater correction ability in both the transverse and sagittal planes when compared with Talar-Fit. Furthermore, he proposed that insertion into the canalis portion would be more beneficial compared with insertion into the sinus portion. Husain (17) studied the biomechanics of STA using screws of different sizes, finding a positive correlation between the size of the screw and the degree of restriction on the subtalar joint, with 6, 8, 9, 10, and 12 mm screws reducing the range of motion by 32.0%, 44.8%, 59.0%, 65.5%, and 76.8%, respectively. Based on the aforementioned information, it can be argued that the selection of implants should take into account the position and size of these implants.

Therefore, the aims of this research are as follows: to present the mid-term outcomes of STA with Talar-Fit implant for treating pediatric flexible flatfoot, to compare the radiographic and clinical outcomes associated with different sizes of Talar-Fit implant, and to analyze the optimal position of the inserted implants.

## 2. Materials and methods

### 2.1. Study design

A retrospective study was conducted on pediatric patients diagnosed with FFF who underwent STA with Talar-Fit (Osteomed, Addison, TX, USA) implant. The study received ethical approval from the Ethics Committee of Huashan Hospital.

### 2.2. Patients

The inclusion criteria for this study were as follows: (a) patients diagnosed with functional and symptomatic FFF and required surgery; (b) patients between the ages of 9 and 18 years at the time of the operation; (c) patients who had undergone STA at Huashan Hospital between January 2014 and December 2021; and (d) patients who had a successful postoperative follow-up of at least 1.5 years.

The exclusion criteria for this study were as follows: (a) patients with rigid flatfoot; (b) patients who had other deformities (neurogenic or neuromuscular disorders); and (c) patients with a history of lower limb surgery or trauma.

A total of 57 children with FFF (77 feet) were finally included in this study. The decision to conduct subgroup analysis was made considering the different sizes of the implants. Consequently, the patients were divided into five groups: Group 8, Group 9, Group 10, Group 11, and Group 12, with the numbers indicating the size of the implant.

### 2.3. Surgical technique

**Figure 1:**
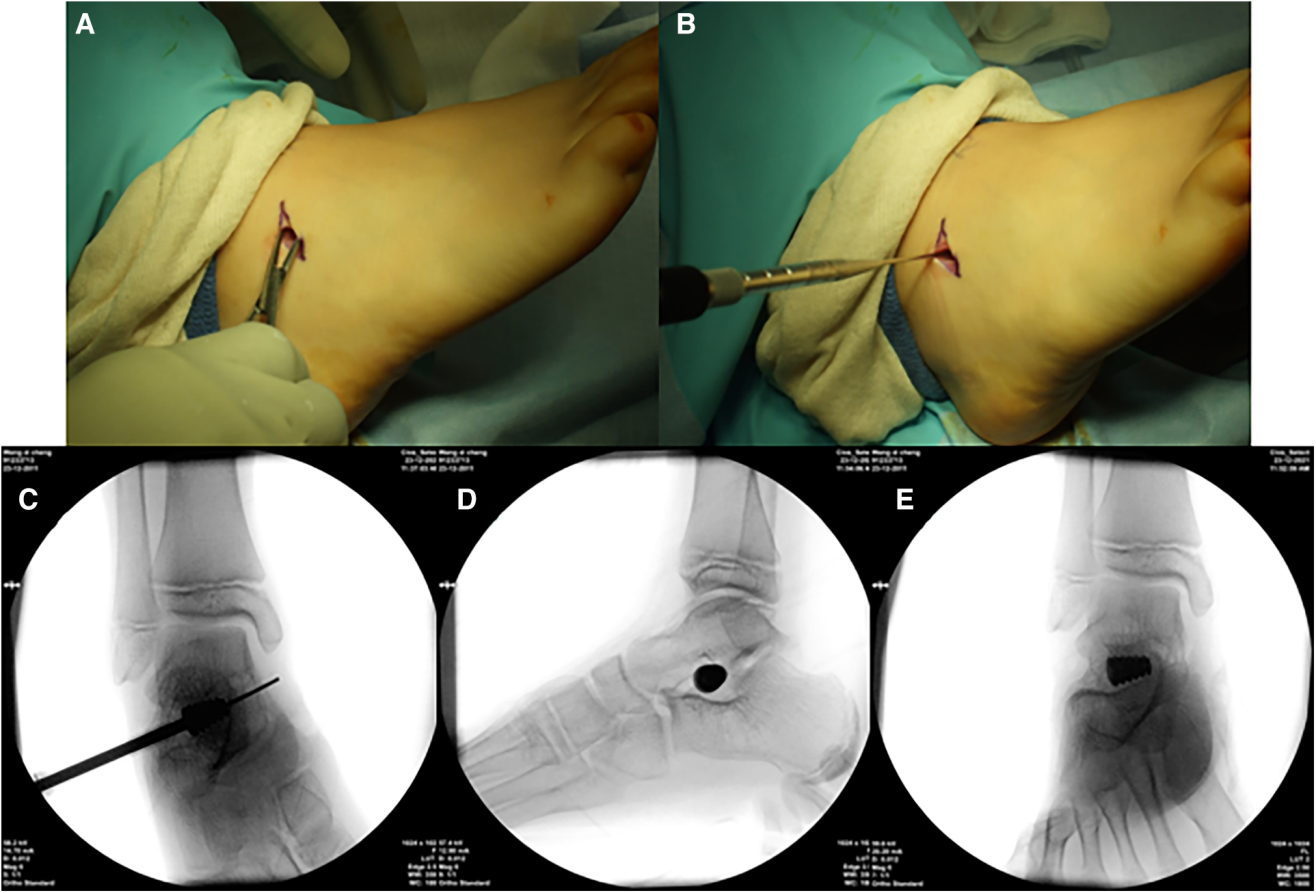
Illustration of some parts of the subtalar arthroereisis. (**A**) Make an incision and expose the tarsal sinus. (**B**) Insert a guide pin and insert the trial implant through the guide pin. (**C**) Check the position of trial implants. (**D,E)** AP and lateral perspectives after the permanent implant was inserted.

The patients were placed in a supine position and underwent surgery while under general anesthesia. A 2 cm incision was made obliquely at the body surface projection of the sinus tarsi. Blunt scissors were utilized to identify the subcutaneous tissue in order to protect the interosseous ligament and superficial nerve branches. Following exposing the tarsal sinus, a guide pin was inserted into the tarsal sinus along the direction of the tunnel. Subsequently, the trial implants were inserted using the guide pin, starting smaller sizes to larger sizes, until the desired hindfoot correction was achieved. The calcaneal axial, weight-bearing AP, and lateral perspectives were examined to check the position of trial implants and evaluate the ability of deformity correction following each implantation. Finally, the screw position and the ability of deformity correction were reevaluated by inserting the most suitably sized implant. The incision was closed layer by layer (Figure 1).

In cases where patients presented with gastrocnemius contracture or Achilles tendon contracture, a simultaneous procedure of gastrocnemius recession (18) or percutaneous Achilles tendon lengthening (19) was performed. Furthermore, in cases where patients exhibit an accessory scaphoid and experience pain at the site of the accessory scaphoid during rest, surgical intervention involving the removal of the accessory scaphoid and simultaneous reconstruction of the posterior tibialis tendon is necessary (20).

A 6-week period of immobilization with a short leg cast was implemented for all patients following the operation. The participants were allowed to engage in functional exercise after 6 weeks.

### 2.4. Outcome assessment

#### 2.4.1. Radiographic outcome

**Figure 2:**
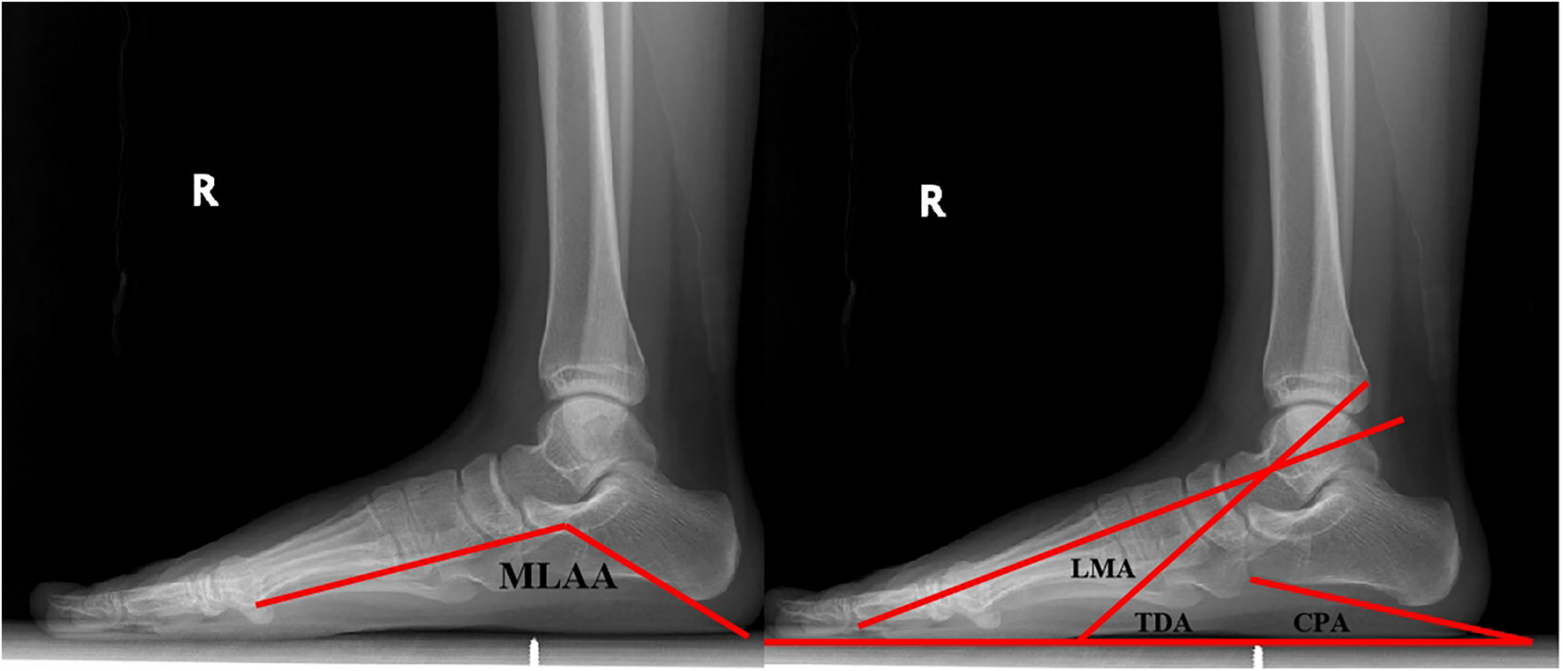
Lateral view of radiograph, demonstrating the measured angles. CPA formed by a line connecting the two most inferior points on the calcaneus and another line parallel to the ground. LMA formed by the axis of the first metatarsal and the longitudinal axis of the talus. TDA formed by a line parallel to the ground and the longitudinal axis of the talus. MLAA formed by a line connecting the most inferior points on the calcaneus and the caput tali and another line connecting the most inferior points on the head of the first metatarsal and the caput tali.

Pre- and postoperative lateral radiographs were performed under weight-bearing in order to assess the surgical outcomes (21). The calcaneal pitch angle (CPA), the lateral Meary angle (LMA), the talar declination angle (TDA), and the medial longitudinal arch angle (MLAA) were measured. The specific measuring methods are displayed in Figure 2.

**Figure 3:**
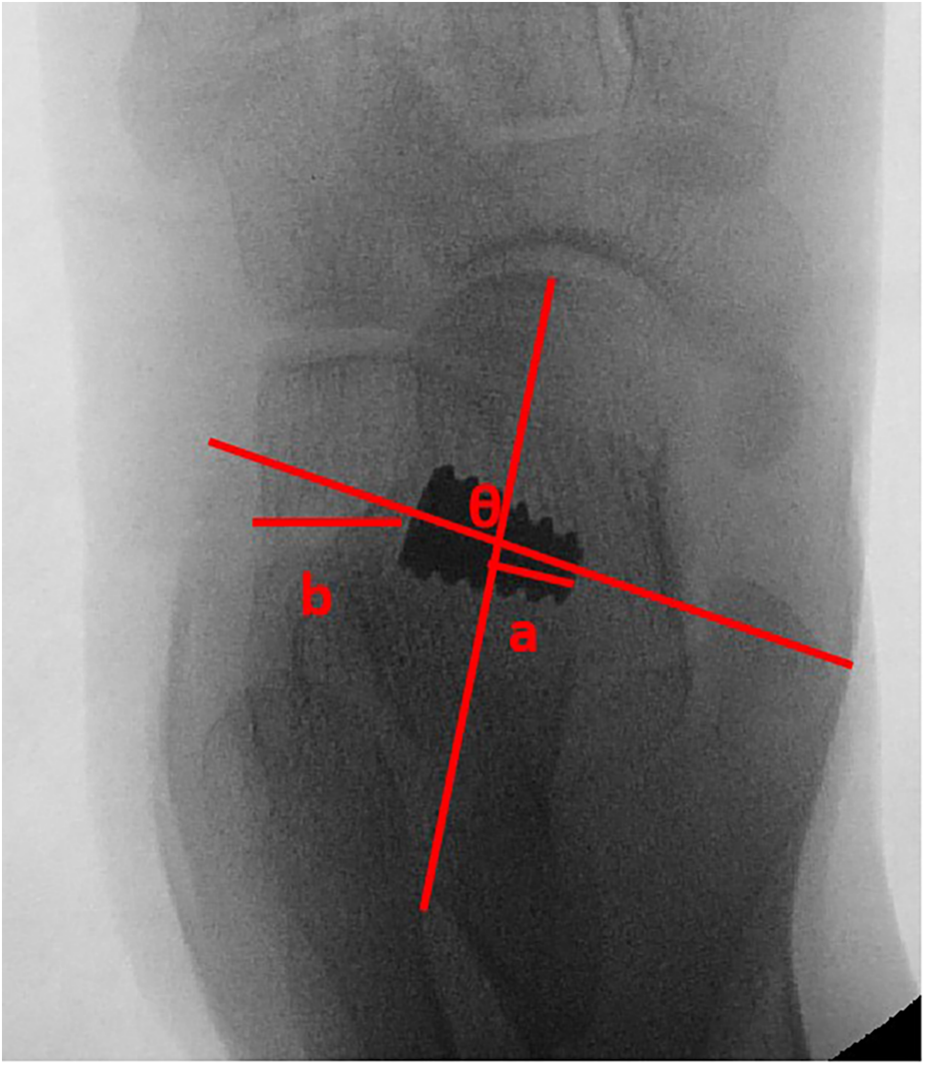
Anteroposterior view of x-ray, demonstrating the position of implant. Angle θ presents the orientation, defined as the angle formed between the talar bisection and the longitudinal axis of implant. Line segment a presents the depth, defined as the perpendicular distance from the medial edge of the implant to the longitudinal talar bisection line. Line segment b presents the distance, defined as the perpendicular distance from the lateral edge of the implant to the lateral calcaneal wall.

An anteroposterior view of postoperative x-ray illustrated the position of implants (22), including depth, orientation, and distance, which are shown in Figure 3.

#### 2.4.2. Clinical outcome

The clinical evaluation was evaluated using the AOFAS ankle–hind foot score, and the postoperative complications were also recorded.

### 2.5. Statistical analysis

The results were presented as the mean and standard deviation. SPSS 17.0 statistical software was utilized for the data analysis. The paired Student's *t*-test was used to compare the AOFAS scores and angular measurements of pre- and postoperative conditions. The Wilcoxon rank-sum test was utilized to analyze the outcome differences among subgroups due to the variations in sample sizes and the non-normal distribution of outcomes within each group. In addition, the sample size of each group was small. Significant differences were defined as *P* < 0.05.

## 3. Result

### 3.1. General characteristics

Among the 57 patients (77 feet) included in our study, 20 (16.1%) were female and 37 (83.9%) were male, with an average age of 13.9 years (range, 11–18). All patients were thoroughly followed-up for a minimum of 18 months, with an average duration of 26.8 months (range, 18–63 months). To estimate the effects of implants with different sizes, the patients were categorized into five groups: Group 8 (*n* = 6), Group 9 (*n* = 15), Group 10 (*n* = 24), Group 11 (*n* = 24), and Group 12 (*n* = 8). The baseline characteristics of different groups were presented in Table 1, and no statistically significant differences were observed among the five groups.

**Table 1:** Comparison of baseline characteristics and outcomes among subgroups.

	Group 8	Group 9	Group 10	Group 11	Group 12	*P*-value
Age (years)	12.5 ± 0.9	13.1 ± 1.2	12.8 ± 0.8	12.1 ± 1.5	13.5 ± 1.1	>0.05
Height (cm)	167.9 ± 10.1	169.5 ± 9.1	170.3 ± 7.9	167.1 ± 11.3	166.1 ± 9.5	>0.05
Weight (kg)	58.6 ± 10.2	56.7 ± 11.2	57.6 ± 13.2	61.3 ± 8.3	60.8 ± 6.8	>0.05
Preoperative						
AOFAS	55.1 ± 12.3	57.6 ± 8.7	61.3 ± 13.8	56.5 ± 19.7	62.8 ± 5.5	>0.05
LMA (°)	20.1 ± 3.8	19.7 ± 4.1	20.7 ± 3.7	20.3 ± 3.4	20.5 ± 2.4	>0.05
CPA (°)	13.6 ± 3.1	14.8 ± 1.3	14.9 ± 1.4	14.8 ± 1.4	15.1 ± 1.1	>0.05
TD (°)	41.1 ± 3.2	40.1 ± 2.2	39.9 ± 2.3	40.9 ± 2.0	40.6 ± 1.4	>0.05
MLAA (°)	141.1 ± 3.8	140.8 ± 2.5	139.8 ± 3.0	139.8 ± 2.5	140.2 ± 1.5	>0.05
Postoperative						
AOFAS	84.9 ± 10.4	86.7 ± 9.1	83.5 ± 7.9	88.3 ± 11.5	85.8 ± 10.5	>0.05
LMA (°)	5.2 ± 1.5	4.8 ± 1.4	4.1 ± 1.4	4.5 ± 1.0	4.7 ± 1.3	>0.05
CPA (°)	20.7 ± 2.7	23.6 ± 2.2	24.1 ± 2.8	23.1 ± 2.1	25.8 ± 1.8	>0.05
TD (°)	29.3 ± 4.3	25.6 ± 3.4	25.1 ± 3.3	25.2 ± 2.0	24.3 ± 1.8	>0.05
MLAA (°)	126.2 ± 5.7	123.0 ± 4.4	121.0 ± 3.0	120.2 ± 2.9	119.8 ± 2.7	>0.05

### 3.2. Clinical and radiographic outcomes

The changes of clinical outcomes were investigated using the AOFAS ankle–hind foot score. It was observed that the mean AOFAS score was 58.6 ± 10.9 prior to the operation, which significantly increased to 85.2 ± 8.6 during the last follow-up (*P* < 0.05). Similar to the radiographic results, all of the angles were changed. Specifically, Meary's angle exhibited a significant decrease from 20.3° ± 3.6° preoperatively to 4.5° ± 1.3° at the last follow-up (*P* < 0.05), the calcaneal pitch angle increased from 14.8° ± 1.61° to 23.6° ± 2.7° (*P* < 0.05), the talar declination angle was also significantly decreased from 40.2° ± 2.3° to 25.5° ± 3.2° (*P* < 0.05), and the medial longitudinal arch angle decreased from 140.1° ± 2.8° to 121.4° ± 3.9° (*P* < 0.05) (Table 2).

**Table 2:** Outcome comparisons between preoperative and last follow-up.

	Preoperative	Last follow-up	*P*-value	Change
AOFAS score	58.6 ± 10.9	85.2 ± 8.6	<0.05	28.3 ± 13.7
LMA(°)	20.3 ± 3.6	4.5 ± 1.3	<0.05	15.8 ± 3.8
CPA(°)	14.8 ± 1.6	23.6 ± 2.7	<0.05	8.8 ± 2.6
TD(°)	40.2 ± 2.3	25.5 ± 3.2	<0.05	14.9 ± 3.5
MLAA(°)	140.1 ± 2.8	121.4 ± 3.9	<0.05	18.7 ± 3.9

### 3.3. Subgroup analysis

Table 3 provides a clear description of the improvements observed in several parameters, including different angles and AOFAS score measured in different groups. On this basis, the effect of different sizes of implants could be evaluated. The study revealed that there was no statistically significant difference in the Meary angle across the five groups, despite improvements in the AOFAS score (*P* > 0.05). However, the calcaneal pitch angle, talar declination angle, and medial longitudinal arch angle exhibited significant differences (*P* < 0.05). However, when doing pairwise comparisons between groups, the adjusted *P*-values were found to be greater than 0.05.

**Table 3:** Comparison of the changes of the outcomes among subgroups.

	Group 8	Group 9	Group 10	Group 11	Group 12	*P*-value
AOFAS	13.5 ± 1.7	14.6 ± 2.8	13.4 ± 1.9	13.5 ± 2.0	12.37 ± 1.3	>0.05
LMA(°)	14.9 ± 3.9	14.9 ± 3.5	16.7 ± 4.2	15.8 ± 3.6	15.8 ± 2.9	>0.05
CPA(°)	11.7 ± 2.4	14.5 ± 3.8	14.8 ± 3.8	15.7 ± 2.9	16.3 ± 2.1	<0.05
TD(°)	6.6 ± 1.3	8.7 ± 2.0	9.2 ± 2.6	8.2 ± 2.5	10.9 ± 2.4	<0.05
MLAA(°)	14.8 ± 4.0	17.8 ± 4.2	18.8 ± 4.4	19.6 ± 3.2	20.3 ± 3.9	<0.05

Table 4 also clearly depicts the position of different sizes of implants. It is evident that the average angle of orientation observed in the entire patient population was 89.5° ± 2.4°, and the depth and distance measurements were 0.9 mm ± 2.1 mm and 9.9 mm ± 0.9 mm, respectively. By further observation, it was determined that there were no significant differences among these five groups on the position of implants.

**Table 4:** Comparison of the position of the inserted implants among subgroups.

	Over all	Group 8	Group 9	Group 10	Group 11	Group 12	*P*-value
Orientation (θ, °)	89.5 ± 2.4	88.4 ± 1.5	88.8 ± 1.7	89.6 ± 1.8	89.8 ± 2.2	90.9 ± 2.1	>0.05
Depth (a, mm)	0.9 ± 2.1	1.38 ± 1.7	1.4 ± 2.1	1.00 ± 2.9	0.8 ± 1.2	−0.03 ± 1.5	>0.05
Distance (b, mm)	9.9 ± 0.9	9.8 ± 1.1	10.1 ± 0.9	9.4 ± 0.7	10.1 ± 0.8	9.7 ± 1.2	>0.05

### 3.4. Complications

Eight patients experienced complications, including six cases of pain occurrence and two cases of implant dislocation (Table 5). Among the six cases of pain occurrence, three patients received conservative treatment in the form of regional block therapy, two patients underwent implant removal after 2 years, and one patient underwent reoperation during the follow-up period. Among the two cases of implant dislocation, one patient was treated non-operatively, while the other underwent implant removal after 2 years.

**Table 5:** Complications among subgroups.

	Group 8 (6)	Group 9 (15)	Group 10 (23)	Group 11 (23)	Group 12 (8)
Pain	1 (16.6%)	1 (6.6%)	2 (8.3%)	2 (8.3%)	0
Dislocation	0	0	0	1 (4.1%)	1 (12.5%)

## 4. Discussion

In recent years, podiatrists have increasingly utilized subtalar arthroereisis in treating pediatric FFF (24). As aforementioned, the concept was first introduced by Chambers in 1946. He inserted autologous bone graft into the tarsal sinus to correct the flatfoot by restricting the excessive eversion of the subtalar joint. In 1952, Grice modified the procedure. A cortical bone graft was used to hold the sinus tarsi open and create extra articular subtalar arthrodesis (8). However, this surgery was no longer adopted due to high incidence of complications such as secondary osteoarthritis. Thus, most of the podiatrists continue to endorse the concept proposed by Chambers, arguing that it is fundamental to restrict the excessive eversion of the subtalar joint without resorting to arthrodesis (23). This provides a direction for the treatment of pediatric FFF. Nowadays, the progressive advancement of technology and materials has facilitated the gradual maturation of subtalar arthroereisis, resulting in the emergence of numerous implant options available in the market. Nevertheless, the use of subtalar arthroereisis continues to be a topic of controversy, particularly with regard to product selection, size choice, and implants positioning.

In recent years, there are lots of reports on subtalar arthroereisis, including biomechanical assessment and clinical follow-up. The majority of scholars believe that STA is beneficial to FFF, which is also confirmed by a retrospective study conducted by Graham et al. (25). By conducting a retrospective analysis of clinical and radiographic data from a sample of 83 adult cases who underwent STA with the implantation of a self-locking wedge type, it was found that most of the patients were satisfied with the functional outcomes of their feet, which exhibited significant changes in relevant angles. Specifically, the mean angle of talar declination and talar second metatarsal decreased by 5.7° and 19°, respectively, whereas the mean angle of calcaneal pitch increased by 0.8°. De Pellegrin et al. (26) investigated the clinical and radiographic outcomes of 485 FFF children treated with the calcaneo-stop implant within 4.5 years, and the result demonstrated that 93.7% of the patients were satisfied, and no complications were reported during the course of the study. The result was also consistent with that of our study, even when the Talar-Fit implant was utilized. In this study, 90.0% of the patients were satisfied with the provided services. The AOFAS ankle–hindfoot score was significantly improved from 58.6 ± 10.9 to 85.2 ± 8.6 during the last follow-up (*P* < 0.05), with related angles changed at different levels.

The selection of the implant size is crucial to this procedure. Insufficient correction of deformity may occur if the implant size is smaller. However, if the implant size is larger, it raises an additional concern regarding the potential for overcorrected deformities. In this regard, different scholars hold different views. Yang pointed out that the principle entails selecting the minimal size that is capable of effectively correcting the deformity and can be stably maintained in the tarsal sinus during the movement of the patients (23). However, according to the study by Wang et al. (15), the selection of implant size should be different. They suggested that selecting the larger option would be the optimal choice, provided that both the smaller and larger options yielded satisfactory outcomes. He insisted that selecting the larger one could decrease the incidence of postoperative sinus tarsi pain. Cook et al. (22) conducted a prospective study, identifying the size of implants as a risk factor of postoperative complications, and also suggested that if both small and large sizes yield satisfactory results, choosing larger sizes may potentially reduce postoperative pain. In addition, another biomechanical assessment in a cadaveric study conducted by Husain (17) reported that the ability of correction increased with the size of implants. In our study, some interesting results were found. It is clear that there were no significant differences observed in the Meary's angle and the AOFAS scores as the size increased. However, the calcaneal pitch angle, the talar declination angle, and the medial longitudinal arch angle were significantly different. As for deeper analysis of pairwise comparisons across groups, the differences were not significant. It was observed that STA not only restricted excessive eversion of the subtalar joint but also raised the head of the caput tali when considering these differences (9). The calcaneus was elevated concurrently with the elevation of the caput tali. Consequently, a bigger size of implants could lead to more obvious elevation of the caput tali. It was recommended to choose the larger option if both sizes were sufficient.

Another key point of this surgery is the positioning of the inserted implants. However, the specific normative standards for Talar-Fit have not yet been definitively established. In addition, there are limited reports available to help guide the management of this surgery. Some researchers believe that implants should be inserted into the tarsal sinus in the direction of the tunnel, with the head of the implant not crossing the midline of the talus neck (23). This study investigated the exact position of Talar-Fit inserted. It was found that the longitudinal axis of the implant was perpendicular to the midline of the talus neck, forming a nearly 90° angle. The angle might be a judgement standard for orientation of the inserted implant. Upon closer observation, the overall depth of the implant was found to be inconsistent with previous reports. As the result showed, the average depth was 0.9 mm ± 2.1 mm. Moreover, the average depths in different groups were 1.38 mm ± 1.70 mm, 1.35 mm ± 2.1 mm, 1.00 mm ± 2.91 mm, 0.76 mm ± 1.23 mm, and −0.03 mm ± 1.5 mm, respectively. A primary observation of depths revealed that the depth decreased as the size increased. However, the results of the statistical analysis did not reveal any significant difference between the groups. The result indicated that each size of Talar-Fit might exceed the midline of the talus neck. Thus, it was hypothesized that the medial edge of the implant might exceed the midline of the talus neck. Further research is still required to determine the extent to which depth can be exceeded, as some researchers consider depth as a risk factor for developing sinus tarsi pain (14, 15, 27, 28).

While this study provides valuable insights, it has some limitations. First, the presence of information bias and selection bias should be acknowledged due to the retrospective design of this study. Second, there was no comparison made between the evaluation of STA and the patients treated with conservation treatment. Third, the sample size was small, resulting in smaller sample sizes across subgroups. A prospective and randomized controlled study should be conducted in the future to confirm our results.

## 5. Conclusions

In conclusion, the findings of our study indicated that subtalar arthroereisis with Talar-Fit implant proved to be an effective treatment for pediatric flexible flatfoot. Furthermore, it was demonstrated that a larger implant size could generate a superior effect compared with the small one. In addition, the present study determined the exact position of Talar-Fit inserted, and indicated that the medial edge of the implant might be able to exceed the midline of the talus neck. Future research can seek to increase the sample size to provide long-term outcomes, and RCTs must be conducted to improve the quality of such studies.

## Data Availability

The original contributions presented in the study are included in the article, further inquiries can be directed to the corresponding author.
